# Varied presentations of congenital ocular synkinesis: do they all fit congenital cranial dysinnervation disorder spectrum?

**DOI:** 10.5935/0004-2749.20210065

**Published:** 2021

**Authors:** Ian Curi, Carlos Souza-Dias

**Affiliations:** 1 Hospital Federal dos Servidores do Estado do Rio de Janeiro, Rio de Janeiro, RJ, Brazil; 2 Instituto Strabos, São Paulo, SP, Brazil

**Keywords:** Synkinesis, Trochlear nerve, Cranial nerves/abnormalities, Oculomotor muscles, Ocular motility disorders/congenital, Sincinesia, Nervo troclear, Nervos cranianos/ anormalidades, Músculos oculomotores, Transtornos da motilidade ocular/congênito

## Abstract

**Purpose:**

Synkinesis results from nerve miswirings and causes aberrant movements of the affected muscles. We present a series of cases of rare congenital ocular synkinesis involving the extraocular muscles and the levator palpebrae superioris and speculate the possibility of classifying these entities in the spectrum of congenital cranial dysinnervation disorder.

**Methods:**

Records of patients with the diagnosis of congenital ocular synkinesis were analyzed retrospectively. We analyzed the sex, laterality, and complete features of the ocular motility of each patient.

**Results:**

Nine patients with congenital ocular synkinesis were included. A slight predominance of women was noted. In terms of laterality, four patients had only the right eye involved, four had only the left eye, and one had both eyes involved. Notably, 55.5% were orthotropic in the primary position. The third, fourth, and sixth cranial nerves were involved in the miswiring in 100%, 44.4%, and 11.1% of the cases, respectively.

**Conclusions:**

Congenital synkinesis might present in a very eclectic and uncommon fashion. The aberrant innervation in these cases classifies them into the group of congenital cranial dysinnervation disorders.

## Abstract (pt)

**Objetivo:**

Sincinesias são resultado de inervações anômalas e ocasionam movimentos aberrantes dos músculos envolvidos. Apresentamos uma série com casos raros de sincinesias oculares congênitas dos músculos extraoculares e do levantador da pálpebra superior e especulamos a possibilidade de classificá-las dentro do espectro das desordens congênitas da desnervação cranianana.

**Métodos:**

Prontuários de pacientes com diagnóstico de sincinesia ocular congênita foram estudados retrospectivamente. Analisamos sexo, lateralidade e as características completas do exame de motilidade de cada paciente.

**Resultados:**

Nove pacientes com sincinesias oculares congênitas foram incluídos. Houve discreta predominância no sexo feminino. Em termos de lateralidade, o olho direito foi o único envolvido em 4 casos, o olho esquerdo também em 4 casos e 1 caso apresentou acometimento bilateral. 55,5% dos pacientes eram ortotrópicos na posição primária. Os III, VI e IV nervos participaram da sincinesia em 100%, 44,4% e 11,1% dos casos, respectivamente.

**Conclusões:**

Sincinesias oculares congênitas podem se apresentar de modo bastante eclético e incomum. A inervação aberrante presente em cada um desses casos os coloca na lista de candidatos a integrar o grupo das desordens congênitas da desenervação craniana.

## INTRODUCTION

Congenital cranial dysinnervation disorders (CCDDs) are non-progressive, sporadic or familial, developmental anomalies of cranial nerves characterized by abnormal eye, eyelid, or facial movements^(1)^. Synkinesis results from aberrant innervation, causing involuntary stimulation of a muscle or structure not normally supplied by that nerve^(2)^. Notably, well-known syndromes like Duane syndrome and Marcus-Gunn jaw winking that demonstrate synkinesis are already part of CCDDs. Because CCDDs is a concept in evolution, several new entities are being described and identified as candidates to be on that list. Notably, some CCDDs are already linked with known genetic abnormalities and well-described image findings, primarily on magnetic resonance.

Herein, we present an eclectic group of patients with rare congenital ocular synkinesis resulting in aberrant movements and speculate the possibility of classifying these patients in the spectrum of CCDDs.

## METHODS

This study was approved by the Review Board of Hospital Federal dos Servidores do Estado do Rio de Janeiro. Both authors retrospectively reviewed the medical records of cases with a diagnosis of congenital ocular synkinesis. However, cases of Marcus-Gunn jaw winking synkinesis, classic Duane syndrome, and other entities that are already established as CCDDs were excluded owing to their higher incidence.

We analyzed the sex, laterality, and complete features of the ocular motility examination of each patient, including the assessment of which cranial nerves and muscles were affected and the deviation from the primary position. In addition, we present pictures emphasizing the primary aspects of the motility disorder and the schematic figures that raise pathophysiological possibilities.

## RESULTS

The study included nine patients with the diagnosis of congenital ocular synkinesis, five women (55.5%) and four men (44.5%). Four patients (44.5%) had only their right eye affected, and four (44.5%) had their left eye affected. Only one (11%) patient had synkinesis in both eyes. The third cranial nerve was involved in all cases, but in four cases (44.5%), this nerve was the only one involved (third to third nerve misdirection). The sixth cranial nerve was involved in the synkinesis in four cases (44.5%) and the fourth nerve in only one patient (11%). Regarding ocular muscles, the levator palpebrae superioris, medial rectus, lateral rectus, and inferior rectus were each involved in four cases. The superior rectus and superior oblique were affected in three cases and one case, respectively. Furthermore, the alignment in the primary position varied as follows: five patients were orthotropic, two patients were esotropic, one exotropic, and one had a combination of exotropia and hypotropia (Table 1).

**Table 1:** Features of the nine cases.

Case	Nerves involved	Muscles involved	Primary position deviation	Laterality	Sex	Concomitant diagnosis
1	III -> VI	SR -> LR	Ortho	RE	M	No
2	III -> VI	SR -> LR	Ortho	LE	F	No
3	VI -> III	LR -> SR	ET	RE	F	No
4	III -> III	MR -> IR	Ortho	LE	M	No
5	VI -> III	LR -> IR	ET	LE	M	No
6	III -> III	MR -> IR	XT	RE	F	Synergistic Abduction
7	IV -> III	SO -> LPS	Ortho	RE	F	No
8	III -> III	MR -> LPS	Otho	OU	F	No
9	III -> III	IR -> LPS	XT + HoT	LE	M	Monocular elevation deficiency

III= third cranial nerve; IV= fourth cranial nerve; VI= sixth cranial nerve; MR= medial rectus; IR= inferior rectus; LR= lateral rectus; SR= superior rectus; LPS= levator palpebrae superioris; Ortho= orthotropic; ET= esotropic; XT= exotropic; HoT= hypotropic; RE= right eye; LE= left eye; OU= both eyes; M= male; F= female.

**Figure 1:**
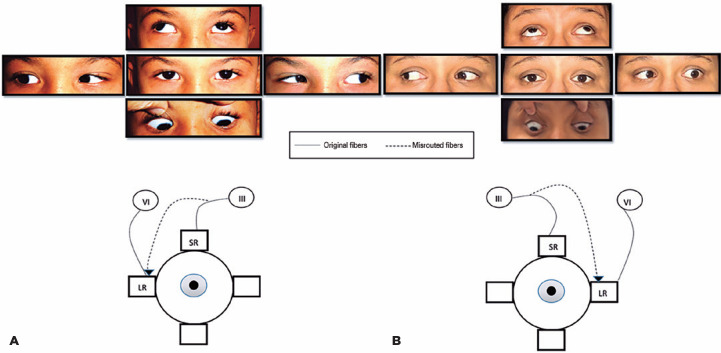
Two cases of dysinnervation between superior rectus and lateral rectus. A) Case 1: Fibers that should go to the right superior rectus innervating the right lateral rectus. Observe the abduction of the right eye in supraduction. B) Case 2: Fibers that should go to the left superior rectus innervating the left lateral rectus in the left eye. Observe the abduction of the left eye in supraduction in addition to the limited abduction of the left eye.

Figure 1 illustrates two patients in whom the lateral rectus received aberrant fibers from the branch of the third nerve that should go to the superior rectus, resulting in anomalous abduction of the affected eye in supraversion. The first one is a male with right eye involvement, and the second one is a female with left eye involvement. Both are orthotropic in the primary position (Figure 1).

**Figure 2:**
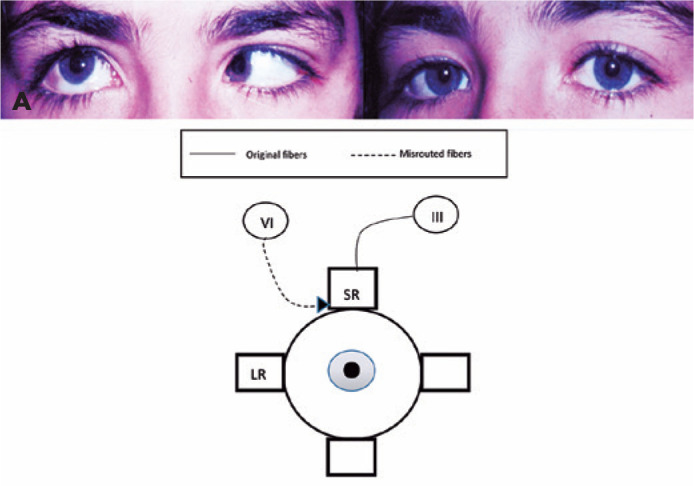
One case of misinnervation between lateral rectus and superior rectus. A) Case 3: Fibers that should go to the lateral rectus innervating the right superior rectus in the right eye. Observe the elevation of the right eye in abduction in addition to the limited abduction of the right eye.

Figure 2 reveals a female with an elevation of the right globe when attempted to abduct. In this case, the misdirection of the sixth nerve fibers compromises the abduction completely, indicating the possibility of anomalous fibers from the sixth nerve innervating the superior rectus in abduction (Figure 2).

**Figure 3:**
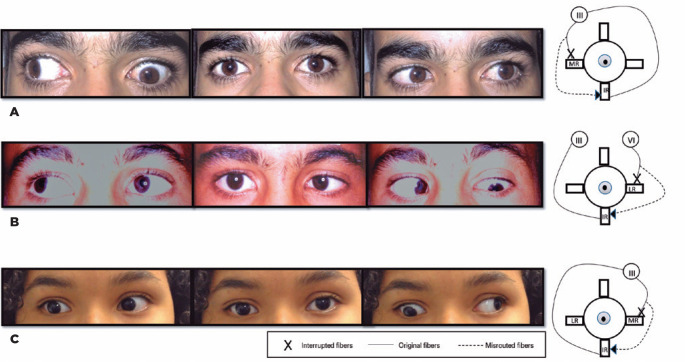
Three cases wherein the inferior rectus receives aberrant innervation.A) Case 4: Fibers that should go to the left medial rectus innervating the left inferior rectus. B) Case 5: Fibers that should go to the left lateral rectus innervating the left inferior rectus. C) Case 6: Fibers that should go to the right medial rectus innervating the right inferior rectus in a patient previously operated for synergistic abduction (miswiring between medial and lateral rectus).

Figure 3 presents three cases with the inferior rectus receiving aberrant fibers. In Figure 3A, in the right gaze, the boy has a depression of the left eye instead of adduction, indicating that the inferior rectus received the fibers that should be directed toward the medial rectus. This dysinnervation resulted in a depression instead of adduction. In Figure 3B, the left eye of a boy depresses when attempted to abduct, leading to the speculation that the inferior rectus receives fibers from the sixth nerve. An esotropia of 40 PD in the primary position was observed. In Figure 3C, the girl previously operated for synergistic abduction (right lateral rectus recession 10 mm) has the same clinical picture as Figure 3A, but in the right eye (*mutatis mutandis*). She has a residual right exotropia of 10 PD, and the synergistic abduction movement vanished with the surgery, with only a persistent right eye depression upon attempted adduction (Figure 3).

**Figure 4:**
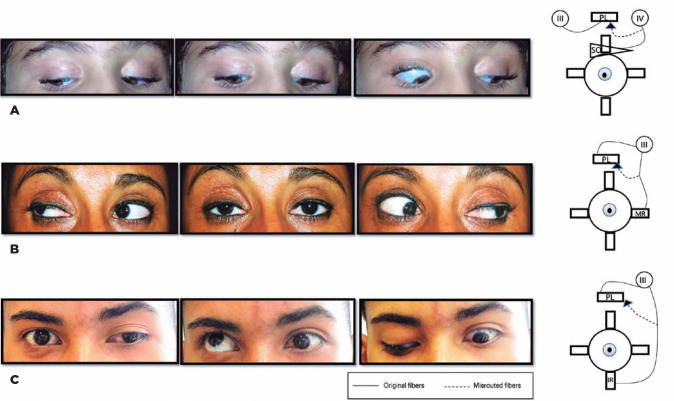
Three cases of miswirings involving the levator palpebrae superioris. A) Case 7: Fibers that should go to the right superior oblique innervating the right levator palpebrae superioris. B) Case 8: Fibers that should go to both medial recti innervating both the levator palpebrae superioris. C) Case 9: Fibers that should go to the left inferior rectus innervating the left levator palpebrae superioris in a patient with ptosis, exotropia, and hypotropia.

Figure 4 presents three cases with eyelid involvement. Figure 4A is a girl who has trochlear-oculomotor synkinesis. Her right levator palpebrae superioris retracted to the diagnostic position of superior oblique muscle (previously reported)^(3)^. Figure 4B is a woman with superior eyelid retraction when adducted bilaterally, indicating that the aberrant fibers that needed to go to both medial recti went to both the levator palpebrae superioris. Finally, Figure 4C is that of a boy with monocular elevation deficiency who has congenital hypotropia and exotropia of the left eye (measuring approximately 10 PD and 30 PD, respectively) and a superior eyelid retraction when looking down. This scenario indicates that the anomalous fibers from the third nerve, which should have gone to the inferior rectus, went to the left eyelid (Figure 4).

Nevertheless, it is crucial to highlight that all the cranial nerve misinnervations described herein were presumed based on the clinical picture. Therefore, each figure contains a schematic representation that speculates the most likely pathophysiological mechanism of the synkinesis.

## DISCUSSION

In 1968, Arthur Jampolsky and Souza-Dias came across a little girl with an unnatural deviation. She had orthotropia in the primary position and infraversion, and a large exotropia in supraversion-similar to cases 1 and 2 in this report. They concluded that the observation could be a new kind of Duane’s syndrome. They speculated that some anomalous fibers of the superior rectus nerve would innervate the lateral rectus, similar to what occurs in Duane’s syndrome. Hence, they decided to call this new clinical picture Duane IV (personal communication). During that point, understanding the bizarre synkinetic movements was at the nascent stage, and CCDD was not a concept yet, and it was not until 2002 that a group of 13 specialists, during the European Neuromuscular Centre Workshop, coined the term CCDD for a group of congenital neuromuscular diseases characterized by abnormal eye, eyelid, or facial movement. This group initially included Duane syndrome, congenital fibrosis of the extraocular muscles, Möbius syndrome, horizontal gaze palsy, congenital ptosis, and congenital facial palsy^(4)^. Previously these conditions were referred to in the literature under various terms, including congenital fibrosis syndromes. The new term chosen reflected the belief that these disorders were neuropathic in origin rather than myopathic and resulted from developmental errors in the innervation of the ocular and facial muscles, and were primarily muscular abnormalities secondary to the dysinnervation.

In strabismology, Duane syndrome is the most common and classic synkinesis, and it is already considered as part of the CCDDs spectrum. In these cases, there is miswiring of fibers from the third nerve to the lateral rectus on attempted adduction, leading to globe retraction and palpebral fissure narrowing. In addition, these patients classically have some degree of limited adduction, abduction, or both. Hence, some authors consider a slight abduction movement deficiency, which some patients presented in this report have (eg., cases 1 and 2), represents mild cases of Duane syndrome^(5)^.

Several rare kinds of congenital ocular synkinesis have been reported before. In a case series regarding ocular synkinesis, Freedman and Kushner highlighted that the abducens nerve is frequently in congenital cases and the oculomotor nerve is sometimes involved in traumatic cases, albeit less frequently than the sixth nerve, and emphasized the absence of any prior reports of miswirings involving the trochlear nerve^(6)^.

In our series, the third nerve was the most involved, followed by the sixth and fourth, respectively. Moreover, a slight predominance of female involvement was noted, similar to Duane syndrome. Both eyes were affected equally, and most patients were orthotropic in the primary alignment and had no concomitant diagnosis. Despite several case reports in the literature^(7-9)^, the lack of large case series regarding congenital ocular synkinesis prevents the comparison of our clinical data. Nevertheless, the present case series will serve as comparative data for future studies.

Notably, all synkinesis cases described here fit the current concept of CCDD because they are all congenital, non-progressive cases of an abnormal eye or eyelid movement. Every new case reported brings a new challenge, once there is a great scientific effort in progress trying to clarify the genetic and molecular basis of CCDDs. It is already known that some CCDDs perturb the gene function critical for correct ocular cranial motor neuron specification, whereas the others perturb gene function necessary for normal axon growth and guidance^(10)^.

Therefore, several CCDD phenotypes, nowadays, have their specific gene loci and gene mutations clarified. It is the case of Duane retraction syndrome (DRS), the most common CCDD, with a reported prevalence ranging from 1:1000 to 1:10,000. The following four DRS genes have been identified to date, each of which causes a small proportion of DRS cases: *MAFB, HOXA1, SALL4*, and *CHN1*^(10,11)^.

Furthermore, several studies involving magnetic resonance imaging are in progress to aggregate information regarding the abnormal cranial nerve development in each phenotype of the CCDDs spectrum^(12,13)^. Notably, it is the absence of the sixth nerve in the DRS type 1 and aplasia of the sixth and seventh cranial nerves most often in patients with Möbius syndrome^(13)^. Unfortunately, during the follow-up period of our patients, the use of magnetic resonance imaging to evaluate CCDDs was not yet widespread, which explains the lack of orbital and brain images in our series.

Nevertheless, it is imperative to describe new phenotypes that can fit the CCDDs spectrum, such as those in this paper, to facilitate scientific evolution. The focus of this paper was to emphasize the clinical pictures of rare congenital ocular synkinesis and to speculate the possibility of incorporating them in the CCDDs group. However, we hope that in the near future, scientific evolution will make molecular, genetic, and imaging diagnosis more accessible, providing patients better diagnosis, and treatment.

## References

[1] Singh A, Pandey PK, Agrawal A, Mittal S K, Rana K. M, Bahuguna C (2016). Congenital cranial dysinnervation disorders. Int Ophthalmol.

[2] Bursztyn LL, Makar I. (2014). Congenital trochlear-oculomotor synkinesis. J Neuroophthalmol.

[3] Curi I. (2019). Trochlear-oculomotor synkinesis: another congenital cranial
dysinnervation disorder?. Arq Bras Oftalmol.

[4] Gutowski NJ, Bosley TM, Engle EC. (2003). 110th ENMC International Workshop: The congenital cranial
dysinnervation disorders (CCDDs) Naarden, The Netherlands, 25-27 October,
2002. Neuromuscul Disord.

[5] Campomanes Eguiarte GA (2000). Síndrome de Duane em Y. Anais do XXII Congreso do CLADE; São Paulo;.

[6] Freedman HL, Kushner BJ. (1997). Congenital ocular aberrant
innervation-newconcepts. J Pediatr Ophthalmol Strabismus.

[7] Khan AO. (2009). A novel form of aberrant innervation in congenital cranial
dysinnervation disorder. J AAPOS.

[8] Pandey PK, Bhambhwani V, Ranjith PC, Kadav M, Aparnaa C. (2016). Congenital third nerve palsy with synergistic depression on
attempted adduction and trigemino-oculomotor synkinesis: Underpinnings of a
spectral dysinnervation disorder. Indian J Ophthalmol.

[9] Sedarous F, Chan TYB, Makar I. (2018). Alternating hypotropia with pseudoptosis: a new phenotype of
congenital cranial dysinnervation disorder. Case Rep Ophthalmol.

[10] Whitman MC, Engle E. (2017). Ocular congenital cranial dysinnervation disorders (CCDDs):
insights into axon growth and guidance. Hum Mol Genet.

[11] Bosley TM, Abu-Amero KK, Oystreck DT. (2013). Congenital cranial dysinnervation disorders: a concept in
evolution. Curr Opin Ophthalmol.

[12] Demer JL, Clark RA, Lim KH, Engle EC. (2007). Magnetic resonance imaging evidence for widespread orbital
dysinnervation in dominant Duane’s retraction syndrome linked to the DURS2
locus. Invest Ophthalmol Vis Sci.

[13] Kim JH, Hwang JM. (2017). Imaging of Cranial Nerves III, IV, VI in Congenital cranial
dysinnervation disorders. Korean J Ophthalmol.

